# Supplementary material for: Sex-specific association between gut microbiome and fat distribution
Source: Nat Commun. 2019 Jun 3;10:2408. doi: 10.1038/s41467-019-10440-5 (PMC6546740; doi:10.1038/s41467-019-10440-5)
Supplement: Supplementary file 1 — Supplementary Information [file 41467_2019_10440_MOESM1_ESM.pdf]

Sex-Specific Association Between Gut Microbiome and Fat Distribution

Min et al.

Supplementary Information

Supplementary Table 1. Association Between Metabolic Syndrome and Regional Fat Mass

		BMI		Android Fat Mass		Gynoid Fat Mass		Android, Gynoid Interaction	
		OR[a]	P-values[b]	OR	P-values	OR	P-values	OR	P-values
Model 1[b]	Male	0.98	0.944	1.54	0.010	-	-	-	-
	Female	0.98	0.923	1.16	0.187	-	-	-	-
Model 2[c]	Male	1.98	0.004	-	-	1.05	0.618	-	-
	Female	1.60	0.004	-	-	0.91	0.118	-	-
Model 3[d]	Male	1.02	0.944	1.57	0.009	0.96	0.699	-	-
	Female	1.19	0.525	1.18	0.91	0.91	0.111	-	-
Model 4[e]	Male	1.04	0.90	1.87	0.049	1.11	0.516	0.99	0.516
	Female	1.24	0.433	2.62	0.020	1.43	0.109	1.000[f]	0.033

[a] OR refers to the odds ratio.
[b] Model 1 only included android fat mass (per 100g) adjusted for age, education, and BMI.
[c] Model 2 only included gynoid fat mass (per 100g) adjusted for the same covariates.
[d] Model 3 included android and gynoid fat mass, adjusted for the same covariates.
[e] Model 4 included android, gynoid fat mass, and their interaction term, adjusted for the same covariates.
[f] The original coefficient is -2.33E-10, after exponentiation it is extremely close to 1.

Supplementary Table 2. Results of the Correlation Tests of Waist-to-Hip Ratio and Regional Fat Distribution

		Correlation Coefficient	95% Confidence Interval	t[a]	df[b]	P-values[c]
WHR[d] & AFR[e]	Total	0.66	(0.57, 0.73)	12.7	210	0.000
	Male	0.50	(0.33, 0.64)	5.6	94	0.000
	Female	0.76	(0.65, 0.82)	11.9	114	0.000
WHR & GFR[c]	Total	-0.82	(-0.86, -0.77)	-20.9	210	0.000
	Male	-0.81	(-0.87, -0.72)	-13.2	94	0.000
	Female	-0.81	(-0.87, -0.74)	-14.9	114	0.000

[a] t refers to the t value from the Pearson's correlation test
[b] df refers to the degrees of freedom of each correlation test
[c] p values are from the Pearson's correlation test
[d] WHR refers to the waist-to-hip ratio
[e] AFR refers to the android fat ratio, GFR refers to the gynoid fat ratio

Supplementary Table 3. Self-Reported Menopause Status and Its Association with Fat Distribution

Among the total of 116 women that were included in the final analysis, 65 of them reported absence of menstruation, 59 were due to menopause, 1 pregnancy, 1 breast feeding, 4 hysterectomy. The rest of 51 women had normal menstruation. None of them had undergone estrogen replacement treatment. The following table summarizes the result of the univariate logistic regressions using android fat ratio and gynoid fat ratio as the independent variable and menstruation status as the outcome.

Association Between Menopausal Status and Overall Obesity and Fat Distribution Measurements in Women (N = 110)						
	BMI		Android Fat Ratio		Gynoid Fat Ratio	
	OR	P-Values	OR	P-Values	OR	P-Values
Model 1[a]	0.95	0.82	-	-	-	-
Model 2[b]	-	-	1.47E-11	0.79	-	-
Model 3[c]	0.97	0.90	5.7E-09	0.86	-	-
Model 4[d]	-	-	-	-	2.6E-02	0.91
Model 5[e]	0.89	0.68	-	-	5.4E-08	0.71

[a] Model 1 only included BMI as the independent variable, menopause status as the outcome, model adjusted for age, education.

[b] Model 2 only included android fat ratio as the independent variable, menopause status as the outcome, model adjusted for age, education. [c] Model 3 included both BMI and android fat as the independent variables, menopause status as the outcome, adjusted for age, education. [d] Model 4 only included gynoid fat ratio as the independent variable, menopause status as the outcome, adjusted for age, education. [d] Model 5 included both BMI and gynoid fat ratio as the independent variable, menopause status as the outcome, adjusted for age, education.

The results indicated that there is no sufficient evidence to support the association between android fat ratio and menopausal status, gynoid fat ratio and the menopausal status.

We further compared the regional fat ratios between men and post-menopausal women. The model adjusted for age, education level, and BMI. The results are summarized as the following:

The Comparison of Regional Fat Ratios Between Men and Post-menopausal Women				
	Android Fat Ratio		Gynoid Fat Ratio	
	Effect Size	P-Values	Effect Size	P-Values
**Model 1[a]**	-0.021	0.000	-	-
**Model 2[b]**	-	-	0.011	0.007
[a] Model 1 tested the differences in android fat ratio, adjusted for age, education, and BMI; men as the reference level. [b] Model 2 tested the differences in gynoid fat ratio, adjusted for age, education, and BMI; men as the reference level.				

Supplementary Table 4. The Correlations Between Android Fat Ratio and Total Fat Mass

To test the correlation coefficients of the android fat ratio and the total fat mass, we applied Pearson's correlation tests in the male and female samples separately. The results are summarized in the following table.

Results from the Pearson's Correlation Tests						
		Correlation Coefficient	95% Confidence Interval	t	df	P-values
**Android Fat Ratio**	**Male**	0.05	(-0.153, 0.247)	0.47	94	0.636
	**Female**	0.20	(0.017, 0.368)	2.17	114	0.032
**Gynoid Fat Ratio**	**Male**	-0.61	(-0.72, -0.47)	-7.5	94	< 0.005
	**Female**	-0.46	(-0.59, -0.30)	-5.6	114	< 0.005

The following figure delineates the shape of the relationship between the android fat ratio and the total fat mass by sex. The shapes of the two fitted local regression smoothing lines share similarities in shape, as indicated in the table above, the correlations in both men and women are unpronounced.

Figure affiliated to Supplementary Table 4

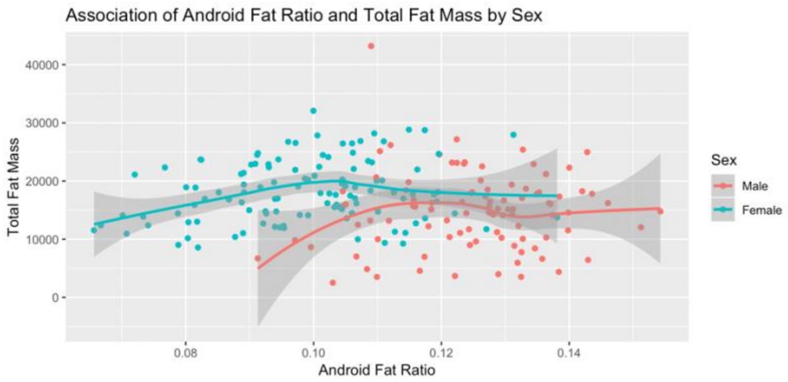

Supplementary Table 5. Linear Association Between BMI, Total Fat Mass, and Height

We constructed a linear model in men and women with BMI as the dependent variable, total fat mass and height as the independent variables. The test result is shown in the following table:

		**Coefficients**	**t value**	**P-values**
**Male**	Total Fat Mass	4.9e-04	22.8	< 0.005
	Height	6.9e-02	-0.05	0.062
**Female**	Total Fat Mass	5.6e-04	28.7	< 0.005
	Height	-1.3e-01	-6.4	< 0.005

The result indicates total fat mass is positively correlated with BMI in both men and women with p-values < 0.005; whereas height is negatively correlated with BMI in both men and women with p-values as 0.062 and < 0.005 respectively. The R^2 for the two models are 0.87 and 0.88 indicating together, total fat mass and height explain 87% of the variance of BMI in men and 88% of the variance in women.

Supplementary Table 6. The correlation between BMI and Fat Ratios in Men and Women

We conducted four correlation tests to investigate the association between BMI and fat ratios (including android & gynoid) in men and women. The testing results are summarized in the table below:

		**Correlation Coefficient**	**95% Confidence Interval**	**t[a]**	**df[b]**	**P-values[c]**
Android Fat Ratio	Male	0.07	(-0.13, 0.27)	0.7	94	0.50
	Female	0.35	(0.18, 0.50)	4.0	114	< 0.005
Gynoid Fat Ratio	Male	-0.66	(-0.77, -0.54)	-8.7	94	< 0.005
	Female	-0.57	(-0.68, -0.43)	-7.4	114	< 0.005

[a] t refers to the t value from the Pearson's correlation test
[b] df refers to the degrees of freedom of each correlation test
[c] p values are from the Pearson's correlation test

Supplementary Table 7. Results of Another Linear Models for Sensitivity Analysis

Table 7-1. Taxa Associated with Android Fat Ratio (Model Excluded Antibiotic Use)*

		Taxa ID	Family	Genus	Log2FoldChange	P-adj
P	Male	ID. 599	Coriobacteriaceae	Eggerthella	7.4	2.2E-03
		ID. 108	Erysipelotrichaceae	Holdemanella	7.4	2.2E-03
		ID. 446	Pasteurellaceae	Haemophilus	7.5	2.2E-03
		ID. 113	Ruminococcaceae	Gemmiger	8.3	5.1E-04
Negative	F	ID. 193	Erysipelotrichaceae	Holdemanella	-9.5	7.5E-05
	Male	ID. 225	Bacteroidaceae	Bacteroides	-7.7	2.2E-03
		ID. 215	Prevotellaceae	Paraprevotella	-9.8	6.3E-06
		ID. 75	Ruminococcaceae	Clostridium_IV	-4.4	8.6E-03

*Adjusted for age, BMI, smoking, alcohol use, dietary fat intake, dietary carbohydrate intake, total energy intake, and sequencing batch

Table 7-2. Taxa Associated with Gynoid Fat Ratio (Model Excluded Antibiotic Use)*						
		Taxa ID	Family	Genus	Log2FoldChange	Padj
P	F	ID.59	Prevotellaceae	Prevotella	10.6	3.9E-03
		ID. 113	Ruminococcaceae	Gemmiger	8.0	7.2E-03
Negative	Female	ID. 271	Lactobacillaceae	Lactobacillus	-6.3	7.3E-03
		ID. 294	Rikenellaceae	Alistipes	-10.8	2.4E-06
		ID. 187	Ruminococcaceae	Ruminococcus	-8.4	3.9E-03
	Male	ID. 114	Bacteroidaceae	Bacteroides	-24.1	2.0E-21
		ID. 93	Bacteroidaceae	Bacteroides	-11.1	1.7E-03
		ID. 214	Lachnospiraceae	Clostridium_XIVa	-5.4	8.7E-03
		ID. 113	Ruminococcaceae	Gemmiger	-10.6	3.0E-03
*Adjusted for age, BMI, smoking, alcohol use, dietary fat intake, dietary carbohydrate intake, and sequencing batch						

Supplementary Figure 1. Sample Sequence Quality Plot

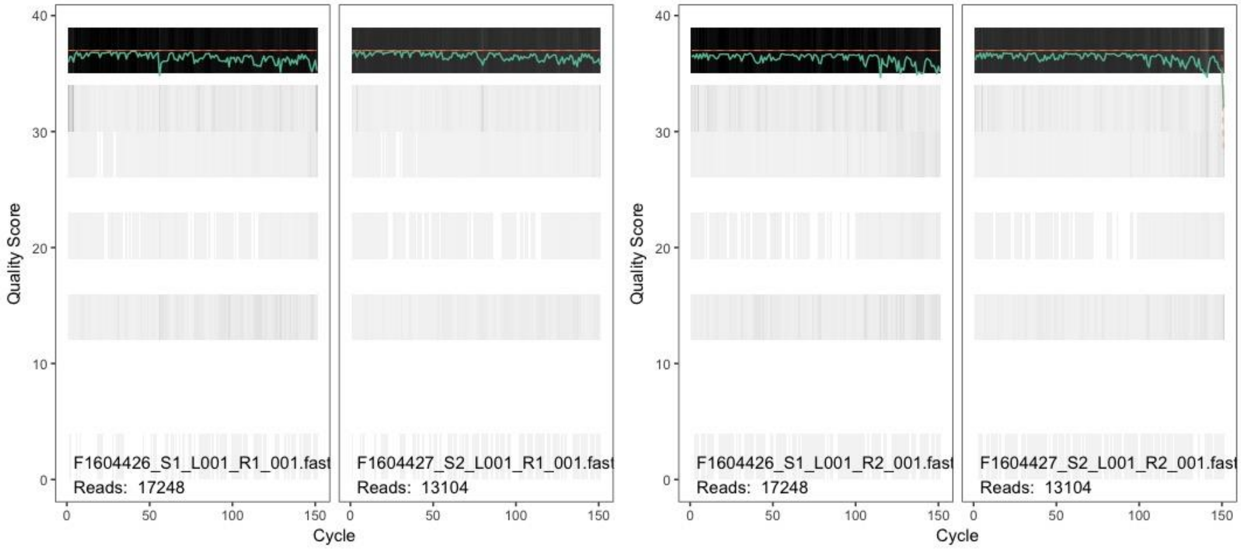

Supplementary Figure 2. Error Rate Plot

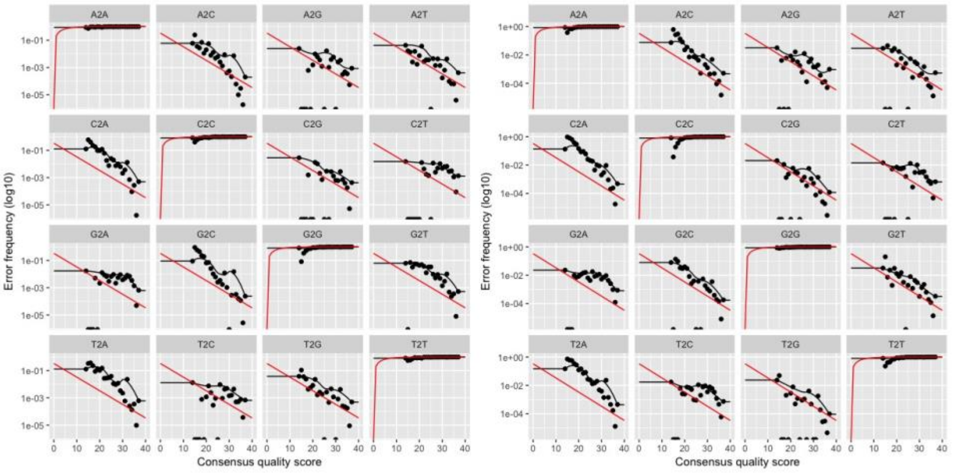

Supplementary Methods. DNA Extraction and 16S rRNA V4 Region Sequencing

DNA was extracted using the MOBIO PowerSoil® DNA Isolation Kit 12888-100 protocol. DNA samples were stored in Tris-EDTA buffer solution at -80 °C. To enable amplification of the genes on the V4 region of the 16S rRNA and add barcode sequences, fusion primers were designed based on the universal primer set: 515F (5’-GTGYCAGCMGCCGCGGTAA-3’) and 806R (5’-GGACTACNVGGGTWTCTAAT-3’), along with barcode sequences. PCR mixtures contained 1 μL of each forward and reverse primer (10 μM), 1 μL of template DNA, 4 μL of dNTPs (2.5 mM), 5 μL of 10 × EasyPfu Buffer, 1 μL of Easy Pfu DNA Polymerase (2.5 U/μL), and 1 μL of double distilled water in a 50 μL reaction volume. Thermal cycling consisted of an initial denaturation step at 95 °C for 5 min, followed by 30 cycles of denaturation at 94 °C for 30 s, annealing at 60 °C for 30 s, and extension at 72 °C for 40 s, with a final extension step at 72 °C for 4 min. Ran amplicons from each sample on agarose gel. Expected band size for 515f-806r is ~300-350 bp. Quantify amplicons with Quant-iT PicoGreen dsDNA Assay Kit (ThermoFisher/Invitrogen cat. no. P11496; follow the manufacturer’s instructions). The amplicon library for high-throughput sequencing on the Illumina MiSeq platform was combined with an equal amount and subsequently quantified (KAPA Library Quantification Kit KK4824) according to the manufacturer's instructions.
