# Supplementary material for: Sex-specific association between gut microbiome and fat distribution
Source: Nat Commun. 2019 Jun 3;10:2408. doi: 10.1038/s41467-019-10440-5 (PMC6546740; doi:10.1038/s41467-019-10440-5)
Supplement: Supplementary file 3 — Reporting Summary [file 41467_2019_10440_MOESM3_ESM.pdf]

nature research

Corresponding author(s): Mike Baiocchi, Shankuan Zhu

Last updated by author(s): 2019/04/18

Reporting Summary

Nature Research wishes to improve the reproducibility of the work that we publish. This form provides structure for consistency and transparency in reporting. For further information on Nature Research policies, see Authors & Referees and the Editorial Policy Checklist.

Statistics

For all statistical analyses, confirm that the following items are present in the figure legend, table legend, main text, or Methods section.

n/a	Confirmed	
☐	☒	The exact sample size (*n*) for each experimental group/condition, given as a discrete number and unit of measurement
☐	☒	A statement on whether measurements were taken from distinct samples or whether the same sample was measured repeatedly
☐	☒	The statistical test(s) used AND whether they are one- or two-sided<br>*Only common tests should be described solely by name; describe more complex techniques in the Methods section.*
☐	☒	A description of all covariates tested
☐	☒	A description of any assumptions or corrections, such as tests of normality and adjustment for multiple comparisons
☐	☒	A full description of the statistical parameters including central tendency (e.g. means) or other basic estimates (e.g. regression coefficient) AND variation (e.g. standard deviation) or associated estimates of uncertainty (e.g. confidence intervals)
☐	☒	For null hypothesis testing, the test statistic (e.g. *F*, *t*, *r*) with confidence intervals, effect sizes, degrees of freedom and *P* value noted<br>*Give P values as exact values whenever suitable.*
☒	☐	For Bayesian analysis, information on the choice of priors and Markov chain Monte Carlo settings
☒	☐	For hierarchical and complex designs, identification of the appropriate level for tests and full reporting of outcomes
☒	☐	Estimates of effect sizes (e.g. Cohen's *d*, Pearson's *r*), indicating how they were calculated

Our web collection on statistics for biologists contains articles on many of the points above.

Software and code

Policy information about availability of computer code

Data collection	No Software has been used in the data collection phase.
Data analysis	All the analysis has been done using an open source software R (version 3.5.1)

For manuscripts utilizing custom algorithms or software that are central to the research but not yet described in published literature, software must be made available to editors/reviewers. We strongly encourage code deposition in a community repository (e.g. GitHub). See the Nature Research guidelines for submitting code & software for further information.

Data

Policy information about availability of data

All manuscripts must include a data availability statement. This statement should provide the following information, where applicable:

- Accession codes, unique identifiers, or web links for publicly available datasets
- A list of figures that have associated raw data
- A description of any restrictions on data availability

The sequencing data that support the findings of this study has been made publicly available at the NIH National Center for Biotechnology Information Sequence Read Archive (SRA) with BioProject ID PRJNA533934, accession codes are available in the supplementary material. The sample data are available from the corresponding author upon reasonable request. The R programming codes related to the statistical analysis are publicly available as part of the supplementary materials.

Field-specific reporting

Please select the one below that is the best fit for your research. If you are not sure, read the appropriate sections before making your selection.

☒ Life sciences ☐ Behavioural & social sciences ☐ Ecological, evolutionary & environmental sciences

For a reference copy of the document with all sections, see nature.com/documents/nr-reporting-summary-flat.pdf

Life sciences study design

All studies must disclose on these points even when the disclosure is negative.

Sample size	The study used cluster sampling. 222 participants were chosen from the all the communities within one subdistrict from the WELL-China Site, which is a longitudinal cohort.
Data exclusions	Ten individuals did not have DXA assessments, did not provide stool samples, or had missing values on covariates were excluded from the study.
Replication	The study has implemented a series of sensitivity analysis to confirm the replication. The results are consistent across the analysis.
Randomization	As the study originated from an observational cohort, randomization is not applicable in this case. Potential confounding has been adjusted in the statistical analysis.
Blinding	As the study is set-up upon an observational cohort, blinding is not applicable in this case.

Reporting for specific materials, systems and methods

We require information from authors about some types of materials, experimental systems and methods used in many studies. Here, indicate whether each material, system or method listed is relevant to your study. If you are not sure if a list item applies to your research, read the appropriate section before selecting a response.

Materials & experimental systems

n/a	Involved in the study	
☒	☐	Antibodies
☒	☐	Eukaryotic cell lines
☒	☐	Palaeontology
☒	☐	Animals and other organisms
☐	☒	Human research participants
☒	☐	Clinical data

Methods

n/a	Involved in the study	
☒	☐	ChIP-seq
☒	☐	Flow cytometry
☒	☐	MRI-based neuroimaging

Human research participants

Policy information about studies involving human research participants

Population characteristics	The final analysis included 212 participants, 96 male and 116 female. The overall mean age is 51 years for both sexes. About 61% of men versus almost 0% of women were current smokers; 70% of men versus 30% women are current alcohol drinkers. The prevalence for metabolic syndrome was 17% in men and 16% in women; the prevalence for type 2 diabetes was 20% in men and 11% in women.
Recruitment	The participants of WELL-China are recruited from 550,000 permanent residents of 12 sub-districts aged 18 – 80 years old. A combination of randomized sampling and quota sampling are applied.
Ethics oversight	The study has obtained the Institution Review Board approvals from both Stanford University (IRB-35020) and Zhejiang University (No. ZGL201507-3).

Note that full information on the approval of the study protocol must also be provided in the manuscript.
